# Shear rate-dependent thrombosis/fibrinolysis tests using non-anticoagulated blood could be useful in the prevention of thrombotic disorders

**DOI:** 10.4155/fsoa-2018-0092

**Published:** 2018-11-19

**Authors:** Junichiro Yamamoto, Yoshinobu Ijiri, Muneshige Shimizu, Shinji Yamamoto, Hideo Ikarugi, Kjell S Sakariassen

**Affiliations:** 1 Kobe Gakuin University (Professor Emeritus), Kobe, Japan; 2 Faculty of Health & Nutrition, Osaka Shoin Women's University, Osaka, Japan; 3 Otsuka Pharmaceutical Company, Saga, Japan; 4 Faculty of Sport Sciences, Nihon Fukushi University, Aichi, Japan; 5 School of Economics, University of Hyogo, Kobe, Japan; 6 Biella, BI, Italy

**Keywords:** diet, exercise, fibrinolysis, native blood, platelet function, shear rate-dependent thrombosis, thrombolysis, thrombus

Prevention of thrombotic disorders such as cardiovascular disease and stroke is an urgent and important task for society. Prevention by suitable diet and exercise is recommended by government guidelines in many countries. In order to get useful and practical results by the recommendations, tests employed to assess the thrombotic status of patients, their quality of diet and their exercise levels have conclusive importance.

Assessment of thrombotic status has been performed over the years by quantifying thrombotic factors and by measuring function using anticoagulated blood samples. However, these approaches do not seem to be successful in assessing thrombotic status. This may be due in part to the belief that adding calcium to anticoagulated blood can restore properties of the original native blood and that individual quantification of thrombotic factors can reflect properties of overall multifactorial native (non-anticoagulated) blood. However, this assumption is wrong and needs to change, not least in particular because relevant *in vivo* wall shear rates are not included in these tests.

A different approach based on physiological (functional) and biological (evolutionary) ideas was proposed by separate groups in the 1970s. The first group was made up of Baumgartner, Sakariassen and colleagues [1–5], and the other of Kovacs and colleagues [6–10]. Both used native blood as samples (*ex vivo*) and thrombosis was measured at various shear rates. The Baumgartner, Sakariassen and colleagues thrombosis tests are triggered by various thrombogenic surfaces, including human arterial subendothelium, human fibrillar collagen and human tissue factor/phospholipids at wall shear rates varying from 100 to 32,000 s^-1^. Blood is drawn directly from an antecubital vein over the prothrombotic surface at various controlled-wall shear rates, thus avoiding coagulation and platelet activation before reaching the prothrombotic surface [4]. These tests have been successfully used in academic and biopharma research since the 1970s. They are used in drug discovery and development and in therapeutic procedures, including optimization of antiplatelet agents and anticoagulation at various wall shear rates [3]. In the Global Thrombosis Tests developed by Kovacs and colleagues, thrombus formation is triggered by high shear stress.

A thrombus is formed by the interaction between blood and blood vessels under blood flow (Virchow's triad). Yamamoto *et al*. introduced the helium–neon laser-induced *in vivo* thrombosis system established by Kovacs *et al*. Subsequently they began research with shear-induced thrombosis/thrombolysis (fibrinolysis) tests both in animals and humans using native blood (*ex vivo*). Yamamoto and colleagues have analyzed the matching results obtained by *ex vivo* and *in vivo* tests in animal experiments. *Ex vivo* and *in vivo* results were closely correlated, although with exception in rodents with severe endothelial dysfunction, recommending a simultaneous endothelial function test (flow-mediated vasodilation test) [11]. No correlation was found between the results obtained by testing diabetics and patients after stroke with a shear-induced thrombosis test using native blood (thrombotic status analyser) and conventional agonist-induced platelet aggregation measurement using anticoagulated blood [12].

Yamamoto *et al*. observed qualitative differences between fruit and vegetable varieties using shear-induced thrombosis/fibrinolysis *ex vivo* tests and He–Ne laser-induced thrombosis *in vivo* test. They demonstrated that the antithrombotic activity of fruits and vegetables varies within the same species, that is, there are varieties with antithrombotic activity, those with prothrombotic activity and those with neither effect [13]. In healthy volunteers, antithrombotic status was observed within 2 h of oral administration of an antithrombotic vegetable variety [14]. Further to this, a daily intake of antithrombotic fruit and vegetable varieties over 12 weeks significantly lowered the thrombotic status of humans [15]. Yamamoto and colleagues proposed that the exercise paradox could be avoided by assessing individual thrombotic status with shear-induced thrombosis/fibrinolysis tests [16]. Similar results have been demonstrated by Sakariassen and colleagues in studies focusing on diet supplement, physical exercise and cigarette smoking [3,17,18].

The *ex vivo* Global Thrombosis Test (GTT) is based on the principles of flow chamber techniques, first described by Baumgartner and Sakariassen, and their colleagues [1,4]. The usefulness of the GTT has already been proven in healthy volunteers [19–21] and in patients [22–25]. Tests using native blood have not been widely used in clinical settings. GTT was first described in 2003 [9] and the first clinical experience with the technique was published by Nishida *et al*. [26]. GTT has been cited in several review articles [27–29]. The slow increase in the number of publications on GTT might be due to the fast coagulation of non-anticoagulated blood, and hence the concern that tests using native blood might not be suitable to assess thrombotic status in clinical settings. Coagulation is, however, slower under the flow conditions in GTT. Previous studies by Baumgartner, Sakariassen, Kovacs and respective colleagues successfully measuring thrombus formation and resolution provided evidence that the use of native blood under flow conditions is superior to studies using anticoagulated blood. As the variability of test results depends on the technique used to obtain the blood sample, there was concern of high variation regarding test results obtained by GTT. However, reproducibility of the GTT has been tested and published [30]. If blood sampling is performed by a trained operator, the reproducibility of both thrombotic (OT) and thrombolytic activity (LT) is good (the intra-assay coefficient of variation of OT was 10% and 6% for LT; the inter-assay coefficient of variation of OT was 8% and 9% for LT), which allows for credible statistical analysis of the findings. The point-of-care GTT has proven to be sensitive in detecting small differences in thrombotic and fibrinolytic activities. GTT surpassed the sensitivity of routine coagulation tests such as the prothrombin time test and the activated partial thromboplastin time test, especially in monitoring oral anticoagulants [31]. GTT detected hyper thrombotic status after overwork, which could not be detected by conventional coagulation tests [32]. Shear-dependent thrombosis tests, including the Baumgartner, Sakariassen and colleagues’ shear dependent tests and the point-of-care *ex vivo* Global Thrombosis Test, could be useful in assessing thrombotic status and bleeding of patients, in developing antithrombotic drugs and diets and in proposing antithrombotic programs utlilizing physical exercise for the prevention of thrombotic disorders.
